# Construction of a tumor-specific bioluminescent eukaryotic expression vector and analysis of its expression *in vitro* and *in vivo*

**DOI:** 10.3892/ol.2013.1343

**Published:** 2013-05-08

**Authors:** QIAN CHEN, FEN LI, FUXIANG ZHOU, WENBO WANG, YU XU, WENJIE SUN, YUNFENG ZHOU

**Affiliations:** Department of Radiation and Medical Oncology, Zhongnan Hospital of Wuhan University, Clinical Cancer Study Center and Key Laboratory of Tumor Biological Behavior, Wuhan, Hubei 430071, P.R. China

**Keywords:** hTERT, tumor-specific vector, telomerase, *in vivo* bioluminescence imaging, stable cell clone

## Abstract

The aims of this study were to construct a tumor-specific bioluminescent eukaryotic vector driven by the hTERT gene promoter and to establish a stable HeLa cell line expressing a modified firefly luciferase gene. PhTERTp-luc and pGL4.17 (luc2/Neo) were digested with *Sac*I and *Hin*dIII, respectively, and the recombinant vector phTERTp-luc-neo was generated by ligating the desired fragments. The expression of phTERTp-luc-neo was tested in a non-transformed cell line (MRC-5), and in telomerase-positive (HeLa, MCF-7 and 293T) and -negative (U2OS and SaOS) transformed cell lines using a luciferase assay. Results showed that the recombinant vector had higher luciferase activity in telomerase-positive transformed cell lines. PhTERTp-luc-neo was transfected into a HeLa cell line, selected by G418 and bioluminescence imaging, and a cell clone HeLa-luc that constitutively expressed both neomycin and luciferase was obtained. We also conducted experiments in animals to observe luciferase activity *in vivo* using stable cell lines that were subcutaneously implanted into BALB/c nude mice and tumor growth was monitored by bioluminescence imaging. The HeLa-luc cell line retained its oncogenicity and tumors were detected on the fifth day following implantation by bioluminescence imaging. This study has formed a basis for the study of the expression and regulation of hTERT and early tumor detection. It also provides a convenient, sensitive and reliable platform for cervical cancer research.

## Introduction

Telomeres are nuclear protein complexes located at the ends of chromosomes, which shorten with cell division. This shortens the telomere and, after 50–70 such divisions (a number known as the Hayflick limit, after its discoverer), a chromosome can grow no shorter and the cell it is in can divide no more. Thus, the cell begins the process of aging, followed by death (1). Telomerase is composed of human Telomerase RNA (hTR), Telomerase-associated protein 1 (TP1) and human Telomerase reverse transcriptase (hTERT). Telomerase is capable of extending or stabilizing the shortened telomeres in the process of cell division by using the subunit hTERT and hTR as a template for synthesizing the telomeric repeat sequence to the ends of chromosomes. Telomerase is important in cell immortalization, and in the occurrence and development of malignant tumors. Positive telomerase expression has been found in 90% of tumor cells, while negative telomerase expression has been identified in the majority of normal human cells (2). Numerous studies (3,4) have indicated that hTR and hTP1 are widely expressed in both tumor and normal tissue. However, hTERT, which is the determined part of telomerase activity, has only been found in the majority of tumors, germ cells and proliferative stem cells (along with its encoded mRNA), and has not been detected in normal tissues (5). Based on these findings, it was concluded that hTERT is important in tumor-specific telomerase activiation. Therefore, how to apply data concerning hTERT activity to the diagnosis and treatment of tumors is the current issue in hTERT research.

*In vivo* bioluminescence imaging technology is a novel type of sensitive optical imaging system. In the present study, cells, proteins or DNA labelled with bioluminescence technology were directly monitored using sensitive optical detection equipment. The movement of cells, protein expression and the genetic behavior of living organisms were monitored *in vivo*. Bioluminescence technology is extremely sensitive, with ∼10^2^ labelled cells having been observed *in vivo* in previous studies that have used this type of technology (6). Compared with the traditional imaging techniques, such as computed tomography (CT) and magnetic resonance imaging (MRI), bioluminescence technology is simple, intuitive, rapid, highly sensitive and inexpensive. Additionally, it is a safe technique that does not require the use of radioactive substances.

The hTERT tumor-specific bioluminescence eukaryotic expression vector constructed in the present study was generated with regard to the bioluminescence imaging system, in order that its expression could be detected in cells and animals. Stable expression of luciferase in the HeLa-luc cell lines was screened for in this study, and the constructed vector was inoculated in nude mice to observe the tumor growth *in vivo*.

## Materials and methods

### Ethics

The present study was approved by the Ethics Review Committee of Zhongnan Hospital of Wuhan University, China.

### Cell culture

Human cervical cancer, HeLa; human breast cancer, MCF-7; human kidney epithelial, 293T and human embryonic lung fibroblast, MRC-5 cell lines were purchased from the Cell Bank of the Chinese Academy of Sciences (Yunnan). Human osteosarcoma cell lines U2OS and Saos were a gift from the Microscopy Orthopedic Laboratory, Research Center of Wuhan University, China.

MCF-7 cell lines were cultured in minimum essential medium (MEM; HyClone Laboratories Inc., Logan, UT, USA) mixed with 10% insulin and 20% fetal bovine serum (FBS). MRC-5 and Saos cell lines were cultured in Roswell Park Memorial Institute (RPMl)-1640 medium (HyClone Laboratories Inc.) mixed with 20% FBS. The remainder of the cell lines were cultured in RPMl-1640 medium mixed with 10% FBS. All the cells were cultured in a humidified incubator at 5% CO_2_ and 37°C.

### Bacteria, plasmids and reagents

*Escherichia coli* (*E. coli*) DH5α was obtained from the Key Laboratory of Virology, College of Life Sciences, Wuhan University, China.

The pGL3 basis of the hTERT promoter was constructed by Dr Liao at the Key Laboratory of Tumor Biological Behaviours, Zhongnan Hospital of Wuhan University, China. PGL4.17(1uc2/Neo) and pGL4.51 (luc2/CMV/Neo) were purchased from Promega Corporation (Madison, WI, USA).

T4 DNA ligase, restriction enzymes *Hin*dIII and *Sac*I and DNA markers were purchased from Takara Bio Inc. (Dalian, China, Japan). Plasmid DNA extraction and gel extraction kits were purchased from TianGen Biotech Co., Ltd. (Beijing, China). A Lipofectamine 2000 kit was purchased from Invitrogen Life Technologies (Carlsbad, CA, USA). The antibiotic, G418, was purchased from Amresco (Solon, OH, USA) and the luciferase substrate was purchased from Kaisheng Medical Technology Co., Ltd. (China).

### Experimental animals

Female BALB/c nude mice (4–6 weeks old) were purchased from the Disease Control Center of Hubei Province (Certificate of Conformity: 0042029), their weights were ∼14–18 g. All the animals were raised in a specific pathogen-free (SPF) environment.

### Construction of recombinant plasmid phTERTp-luc-neo

Restriction enzymes *Sac*I and *Hin*dIII were used to digest plasmid pGL3 basic hTERT promoter to pGL4.17 (luc/neo) and run in gel electrophoresis. The recovered fragments of phTERTp-luc-neo and pGL4.17 from gel extraction were then connected with T4 ligase (4°C overnight), transformed into DH5α, and then the plasmid was extracted from selected clones using a plasmid DNA extraction kit. Preliminarily the plasmid was identified by electrophoresis after double digestion (*Sac*I and the *Hin*dIII), and then the identified plasmid was sequenced (sequenced by invtrogen company).

### hTERT promoter expression detection in vivo

Telomerase-positive (HeLa, MCF-7 and 293T), telomerase-negative (U2OS and SaOS) and normal human embryonic lung (MRC-5) cell lines were separately seeded in 24-well plates, with each cell line being inoculated with 12 holes. Each group was inoculated with three holes following transfection with the recombinant vector phTERTp-luc-neo, positive control vector pGL4.51 (luc2/CMV/Neo) and negative control vector pGL4.17 (luc2/ neo). A blank control was set up for non-transfected plasmids. After 48 h of transfection, the cells were digested to the state of suspension (100 *μ*l per well), and then transferred to 96-well cell plates. One microliter of 15 mg/ml luciferase substrate was added to each hole, mixed and incubated at 37°C for 5 min. Images were then captured using the *in vivo* bio-optical imaging system (Kai Sheng Branch *in vivo* bioluminescence imaging optical system), white light imaging for 0.1 sec and fluorescence imaging for 1–3 min. Bioluminescence intensity was recorded for each cell line.

### Stable transfection with hTERTp-luc-neo

HeLa cells were adjusted to 10,000/ml for detection of the minimum lethal concentration of G418 in the HeLa cell line, and 0.5 ml/well was added to the 24-well plates. Eight concentrations (300, 400, 500, 600, 700, 800, 900 and 1000/ml) of G418 were used for the selection of HeLa cell lines, with each concentration added to three wells. The minimum concentration in which all the cells had died after 10–14 days was selected for screening of HeLa cells.

The logarithmic phase of HeLa cells for recombinant vector transfection and monoclonal screening was selected, and seeded into 6-well culture plates (2×10^5^ cells/well) 24 h prior to transfection. Transfection was conducted according to the manufacturer’s instructions for the Lipofectamine 2000 Kit. G418 was used to screen for the optimal concentration 24 h after transfection, and monoclonal cell lines were screened with a limited cloning dilution method when there was no futher cell death.

Suspension (10^7^ cells/ml) with the initial screening of monoclonal cell lines was generated to identify the positive clone by the *in vivo* bioluminescent imaging system, by adding 100 *μ*l/well to the 96-well plates, with three wells per group. Luciferase substrate (1 *μ*l; 15 mg/ml) was added to each well and mixed for 5 min at 37°C. Images were then captured using the *in vivo* bioluminescence imaging system.

The selected screened positive monoclonal (HeLa-luc) cells and the HeLa cells that were used as a control for the single cell suspension were then added to 24-well plates at 2×10^4^ cells/well. The following day, cells (per three wells) were digested and counted. The cell doubling time (t_D_) was calculated over the subsequent six consecutive days, using the formula: t_D_ = t × lg2 / lg (N/N_0_) (t, time interval in hours; N_0_, cell number at start; N, cell number in the end. The experiment was repeated three times and a cell growth curve was generated.

The screened monoclonal cells were diluted in a number of gradient concentrations to determine the fluorescence value of each gradient concentration, with cell counts of 10^6^, 2×10^5^, 10^5^ and 10^4^, per 100 *μ*l medium. The image was then captured by the *in vivo* bioluminescence imaging system. The bioluminescence intensity of each clone was compared, and the correlation between bioluminescence intensity and the cell number was analyzed. The cell line which demonstrated the highest correlation and the highest high luciferase activity was selected for determination of the fluorescence value of the cell line in various gradient concentrations.

### In vivo observation stably expressing luciferase tumor cell growth

The logarithmic growth phase of the screened monoclonal cells was selected, and the concentrations were adjusted with phosphate-buffered saline (PBS) to 10^4^, 10^5^, 10^6^ and 10^7^ cells/ml. Gradient concentrations of cell suspensions (100 *μ*l) were implanted subcutaneously into each side of the dorsal axillary and groin regions of a nude mouse, at a total of 4 points. In another nude mouse, 10^7^ cells were subcutaneously inoculated into one side of the dorsal axillary region. The mice were administered an intraperitoneal luciferase substrate injection (150 mg/kg) 5–6 min prior to imaging. Subsequently, the mice were administered an intraperitoneal 1% pentobarbital sodium injection (100 mg/kg) during imaging. The duration of the imaging process was 1–3 min.

### Statistical analysis

Experimental data were recorded as mean ± standard deviation. Data were analyzed by a Dixon’s Q test using the Statistical Package for the Social Sciences (SPSS) software, version 13.0. P<0.05 and P <0.01 were used to indicate statistically significant differences.

## Results

### Restriction endonuclease

**Figure 1.**
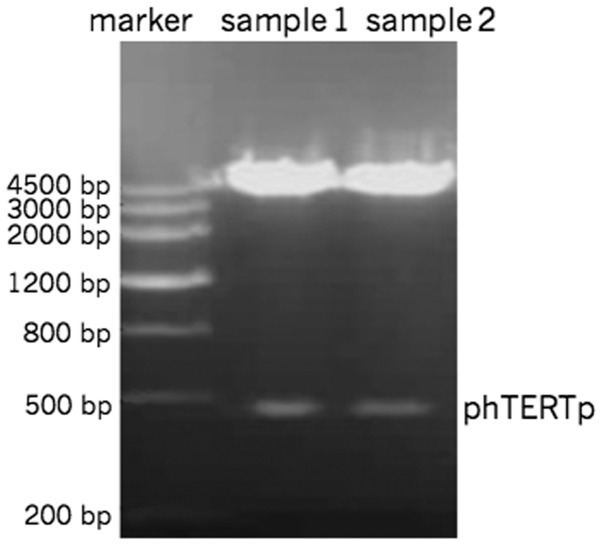
PhTERTp-luc and pGL4.17 (luc2/Neo) were separately digested with restriction enzymes *Sac*I and *Hin*dIII, and the recombinant vector (PhTERTp-luc-neo) was generated by connecting the desired fragments. PhTERTp-luc-neo was verified by double enzyme digestion and sequencing. Clear visible bands of the digestion products at 500 bp by electrophoresis are shown.

PhTERTp-luc-neo was double-digested with *Sac*I and *Hin*dIII. The digestion products revealed clear bands at 500 bp by gel electrophoresis (Fig. 1). The hTERT promoter sequence was identical to that of Genbank.

### Expression of the hTERT promoter following transient transfection

**Figure 2.**
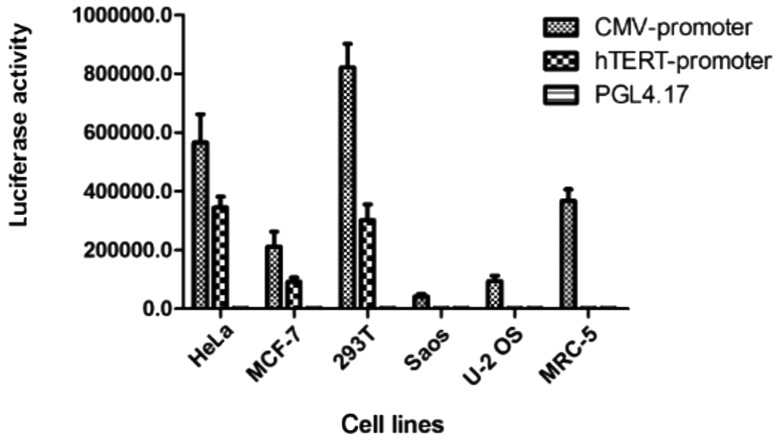
Expression of the phTERTp-luc-neo construct was analyzed in a non-transformed cell line (MRC-5), and in telomerase-positive (HeLa, MCF-7 and 293T) and -negative (U2OS and SaOS) transformed cell lines, using a luciferase assay. The recombinant vector demonstrated a higher luciferase activity in the telomerase-positive transformed cell lines.

Significant luciferase expression was demonstrated by pGL4.51 (luc2/CMV/Neo) in each cell line, although this expression was not tumor-specific. The expression activity of luciferase regulated by hTERT in the telomerase-positive cell lines (HeLa, MCF-7, U251 and 293T) was significantly higher than that of the telomerase-negative (U2OS and Saos) and normal (MRC-5) cell lines. This result confirmed that the constructed vector, phTERTp-luc-neo, was tumor-specific (Fig. 2).

### Identification of positive clones

The optimal concentration of G418 selection in HeLa cells was determined to be 800 *μ*g/l by the G418 gradient concentration filter.

### Initial screening of 30 clones

The transfected plasmid vector was randomly integrated into the chromosome, according to the luciferase activity of the different clones. The luciferase-expressing clones 2, 6 and 15 were determined to be the positive clones and were designated as HeLa-luc-2, -6 and -15, respectively.

### Doubling time of clone cells cultured in vitro

According to the growth curves of the positive clonal cells, HeLa-luc-2, -6 and -15, the doubling times were 28.41, 22.37 and 30.20 h, respectively, whereas the doubling time of HeLa control cells was 22.11 h. We found that the growth of clonal HeLa-luc-6 cells was similar to that of HeLa control cells; no significant difference was observed (P>0.05).

### Determination of the cell fluorescence value of the gradient concentration in vitro

**Figure 3.**
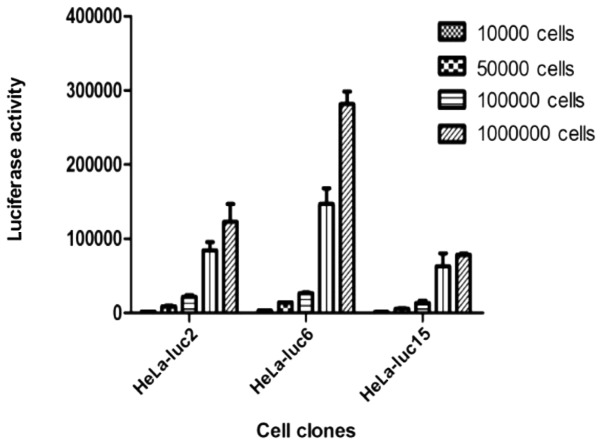
The recombinant vector, phTERTp-luc-neo, was transfected into a HeLa cell line. Antibiotic G418 was used to select neomycin-positive cell clones. Bioluminescent imaging was applied to screen and verify whether selected neomycin-positive cell clones exhibited specific, high luciferase expression. A HeLa-luc cell clone, which constantly expressed both neomycin and luciferase, was obtained by selection with G418 and bioluminescent imaging. According to the luciferase activity of the different clones, the lucif-erase-expressing clones 2, 6 and 15 were determined to be the positive clones, and were termed HeLa-luc-2, -6, and -15, respectively. Cell fluorescence values of the four gradient concentrations (10^4^, 5×10^4^, 1×10^5^ and 1×10^6^ cells/100 *μ*l) were determined. Data showed that the highest correlation between the cell number and fluorescence was in the HeLa-luc-6 monoclone; the correlation coefficient was 0.9937 and an extremely high luciferase activity was observed.

Cells were diluted to four gradient concentrations (10^4^, 5×10^4^, 1×10^5^ and 1×10^6^ cells/100 *μ*l) and cell fluorescence values of the four gradient concentrations were determined. The data showed that there was a higher correlation between the cell number and fluorescence in the HeLa-luc-6 monoclone; the correlation coefficient was 0.9937 and an extremely high luciferase activity was observed (Fig. 3).

### Determination of cell fluorescence of HeLa-luc 6 in gradient concentration

**Figure 4.**
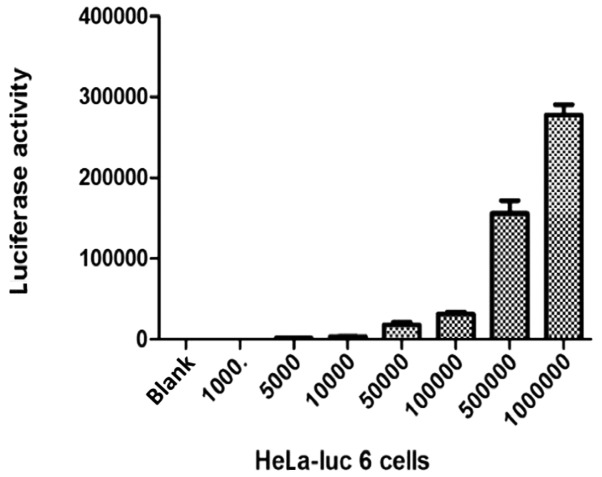
Bioluminescent imaging was applied to screen for and verify the specific luciferase expression of HeLa-luc-6. Cells were diluted into seven gradient concentrations (1000, 5000, 10^4^, 5×10^4^, 1×10^5^, 5×10^5^ and 1×10^6^ cells/ml) and cell fluorescence values of the different gradient concentrations were determined.

The cells were diluted into seven gradient concentrations (1000, 5000, 10^4^, 5×10^4^, 1×10^5^, 5×10^5^ and 1×10^6^ cells/ml). Cell fluorescence values of the seven gradient concentrations were subsequently determined (Fig. 4).

### Animal experiments

**Figure 5.**
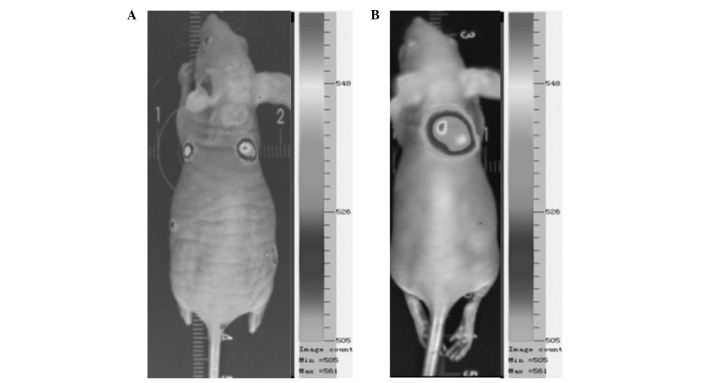
BALB/c nude mice were subcutaneously inoculated with stable HeLa-luc-6 cell lines and tumor growth was monitored using bioluminescent imaging. The HeLa-luc cell line retained its oncogenicity; detection of the tumor on the (A) fifth and (B) twentieth days after implantion by bioluminescent imaging is shown.

Tumor growth was clearly visible subcutaneously in the mouse implanted with HeLa-luc-6 cells, and the tumor was detected on the fifth day following implantation by bioluminescent imaging, demonstrating that the HeLa-luc-6 cells were tumorigenic. During imaging of the mouse subcutaneously implanted with HeLa-luc-6 cells, the expression of luciferase was clearly detected (Fig. 5).

## Discussion

The aim of the present study was to develop methods for testing the usage of *in vivo* bioluminescence imaging technology in tumor detection, for this purpose. Therefore, we constructed the hTERT tumor-specific bioluminescence eukaryotic expression vector and established a stable HeLa-luc cell line that expressed the luciferase gene. Nude mice were then inoculated with the constructed vector in order to observe the tumor growth *in vivo*.

The specific promoter was selected to be 480 bp located at the proximal region of the hTERT gene. The eukaryotic expression vector phTERTp-luc-neo was constructed and was regulated by the hTERT promoter. The recombinant vector was characterized by this in that the hTERT promoter regulated the expression of the downstream luc gene, and showed highly specific expression in telomerase-positive tumor cells. In addition, its expression was observed both *in vitro* and *in vivo* using *in vivo* bioluminescence imaging technology.

The bioluminescence generated in cells labeled with the vector, on addition of the luciferase substrate, was detected by a charge-coupled device (CCD) camera without the existence of an exogenous excitation light source.

Both the experimental and control plasmids were transiently transfected into telomerase-positive, -negative and normal cell lines by lipofection. The tumor-specific characteristic of the plasmid was confirmed by detecting the fluorescence intensity with the *in vivo* bioluminescence imaging system. The i*n vivo* bioluminescence imaging system was found to be more intuitive and convenient than dual luciferase reporter gene detection for the detection of luciferase gene expression. The *in vivo* bioluminescence imaging technology is predicted to become increasingly applied to the field of cancer research.

A study by Jenkins *et al* applied the *in vivo* bioluminescence imaging technique to the observation of tumor cell growth and metastasis (7). Additionally, Gupta investigated breast cancer metastasis, gene expression and the tumor microenvironment by an *in vivo* bioluminescence imaging technique (8). The high sensitivity of *in vivo* bioluminescence imaging technology has been widely used to construct tumor models (9–11); however, its usage in combination with the hTERT promoter in tumor diagnosis and gene therapy is not common. In the present study, the tumor-specific eukaryotic expression vector, phTERTp-luc-neo, an integration of the luciferase reporter and neo genes, was transfected into HeLa cells by lipofection. The transfected cells were monoclonal due to the implementation of a limited cloning dilution method a period of time after G418 selection, to ensure that the cells were obtained from the same ancestors, and that the genetic traits were consistent. Luciferase expression of monoclonal cells was then detected by an *in vivo* bioluminescence imaging system. Three cell lines demonstrated luciferase expression. Dilution of cells to gradient concentration and determination of luciferase expression of each gradient concentration, were conducted, and HeLa-luc-6 cells were selected for further animal experiments. BALB/c mice were subcutaneously inoculated with HeLa-luc-6 cells. The results showed that the cells were tumorigenic and the bioluminescence signal was detected.

In summary, we constructed a tumor-specific bioluminescence eukaryotic expression vector regulated by an hTERT promoter. The vector was visual, intuitive and highly sensitive, and demonstrated potential for the study of gene therapy with telomerase or hTERT as the target. In addition, HeLa-luc cell lines that stably expressed luciferase were established. This study has provided an intuitive, convenient, sensitive and reliable basis for investigation of the expression and regulation of hTERT, and with the early diagnosis of tumors. It also promotes the use of the *in vivo* bioluminescence imaging technique in subsequent experiments.
